# High normal alanine aminotransferase is an indicator for better response to antiviral therapy in chronic hepatitis B

**DOI:** 10.3389/fimmu.2024.1367265

**Published:** 2024-03-14

**Authors:** Chao Cai, Wen-Xuan Shang, En-Hua Lin, Yu-Chun Jiang, Hong Chen, Ke Xu, Lu Chen, Rui-Cong Chen, Yi-Jing Cai, Ji Lin, Ting-Chen Cai, Xiu-Li Lin, Lei Zhang, Nai-Bin Yang, Hui-Fang Zhang, Ming-Qin Lu

**Affiliations:** ^1^ Department of Infectious Diseases, The First Affiliated Hospital of Wenzhou Medical University, Wenzhou, Zhejiang, China; ^2^ Department of Information Technology (IT), The First Affiliated Hospital of Wenzhou Medical University, Wenzhou, Zhejiang, China; ^3^ Department of Gastrointestinal Surgery, National Key Clinical Specialty (General Surgery), The First Affiliated Hospital Of Wenzhou Medical University, Wenzhou, Zhejiang, China; ^4^ Department of Infectious Diseases, Ningbo First Hospital, Ningbo Hospital of Zhejiang University, Ningbo, Zhejiang, China

**Keywords:** chronic hepatitis B, high-normal alanine aminotransferase, antiviral therapy, liver biopsy, histological disease, HBV DNA clearance, predict model

## Abstract

**Background:**

Evidence shows people living with CHB even with a normal ALT (40U/L as threshold) suffer histological disease and there is still little research to evaluate the potential benefit of antiviral benefits in them.

**Methods:**

We retrospectively examined 1352 patients who underwent liver biopsy from 2017 to 2021 and then obtained their 1-year follow-up data to analyze.

**Results:**

ALT levels were categorized into high and low, with thresholds set at >29 for males and >15 for females through Youden’s Index. The high normal ALT group showed significant histological disease at baseline (56.43% *vs* 43.82%, p< 0.001), and better HBV DNA clearance from treatment using PSM (p=0.005). Similar results were obtained using 2016 AASLD high normals (male >30, female >19). Further multivariate logistic analysis showed that high normal ALT (both criterias) was an independent predictor of treatment (OR 1.993, 95% CI 1.115-3.560, p=0.020; OR 2.000, 95% CI 1.055-3.793, p=0.034) Both of the models had higher AUC compared with current scoring system, and there was no obvious difference between the two models (AUC:0.8840 *vs* 0.8835)

**Conclusion:**

Male >30 or female >19 and Male >29 or female>15 are suggested to be better thresholds for normal ALT. Having a high normal ALT in CHB provides a potential benefit in antiviral therapy.

## Introduction

Viral hepatitis B is a major problem affecting the public health of the world. According to the report by the World Health Organization in 2017, about 300 million people worldwide are infected with hepatitis B virus, causing about 800,000 deaths each year (1). Hepatitis B virus infection is not directly cytopathic, the host’s response to virus-infected hepatocytes mediate hepatocyte damage. Long-term chronic liver inflammation leads to the progression of chronic hepatitis B (2).

According to the existing research and treatment experience, the natural course of chronic hepatitis B is usually divided into four stages: immune tolerance stage, immune clearance stage, inactive stage, and reactive stage (3–5). However, they do not cover the whole process of HBV infection, a grey zone exists (**Additional file 1**). Many clinical studies have reported a high proportion of patients belonging to Grey Zone (27.8% - 81.8%) and indicating that the Grey Zone is not a transitional state between the stages of the natural course of hepatitis B (6–9). More importantly, patients in the Grey Zone have a significant risk for morbidity and mortality (10).

It seems the current antivirus strategy needs to be changed, starting with the upper limit of ALT. The 2016 guidelines of the Asia Pacific Association for the study of the Liver (APASL) recommend that 40U/L be used as the upper limit of the normal value of ALT (3). Both the 2007 and 2016 American Association for the Study of Liver Diseases (AASLD) Hepatitis B guidelines recommend that the normal threshold for ALT should be set at 30U/L for male and 19U/L for female. While the 2018 AASLD recommended to use 35U/L (male) and 25U/L (female) as the upper limit of the normal value of ALT in clinical practice. Additionally, the complexity and strictness of current clinical practice guidelines make it difficult to identify which patients can benefit from antiviral treatment. Recommendations are difficult to implement in real-world. The Chinese Prevention and treatment of CHB guideline (2019/2022) both recommend using antiviral therapy in people with positive HBV DNA when ALT continues to exceed the ULN and other causes are excluded or with family history of HCC or liver fibrosis/cirrhosis (11, 12). But the ALT UNL is still 40U/L. At the meeting of the expanding CHB treatment held by the American Hepatitis B Foundation in November 2022, it was proposed that the criteria for the treatment of CHB are only one: HBV DNA-positive, no matter what the ALT value is. However, there is little research focused on the antiviral effect of people with an ALT level< 40U/L.

To identify who can benefit from antiviral treatment from those who do not need treatment according to the current guidelines we retrospectively studied the CHB in people with ALT<40U/L who underwent liver biopsy in 2017 to 2021 and followed those taking antiviral treatments to establish a model to predict HBV DNA clearance rate after antiviral treatment.

## Materials and methods

### Patients

**Figure 1:**
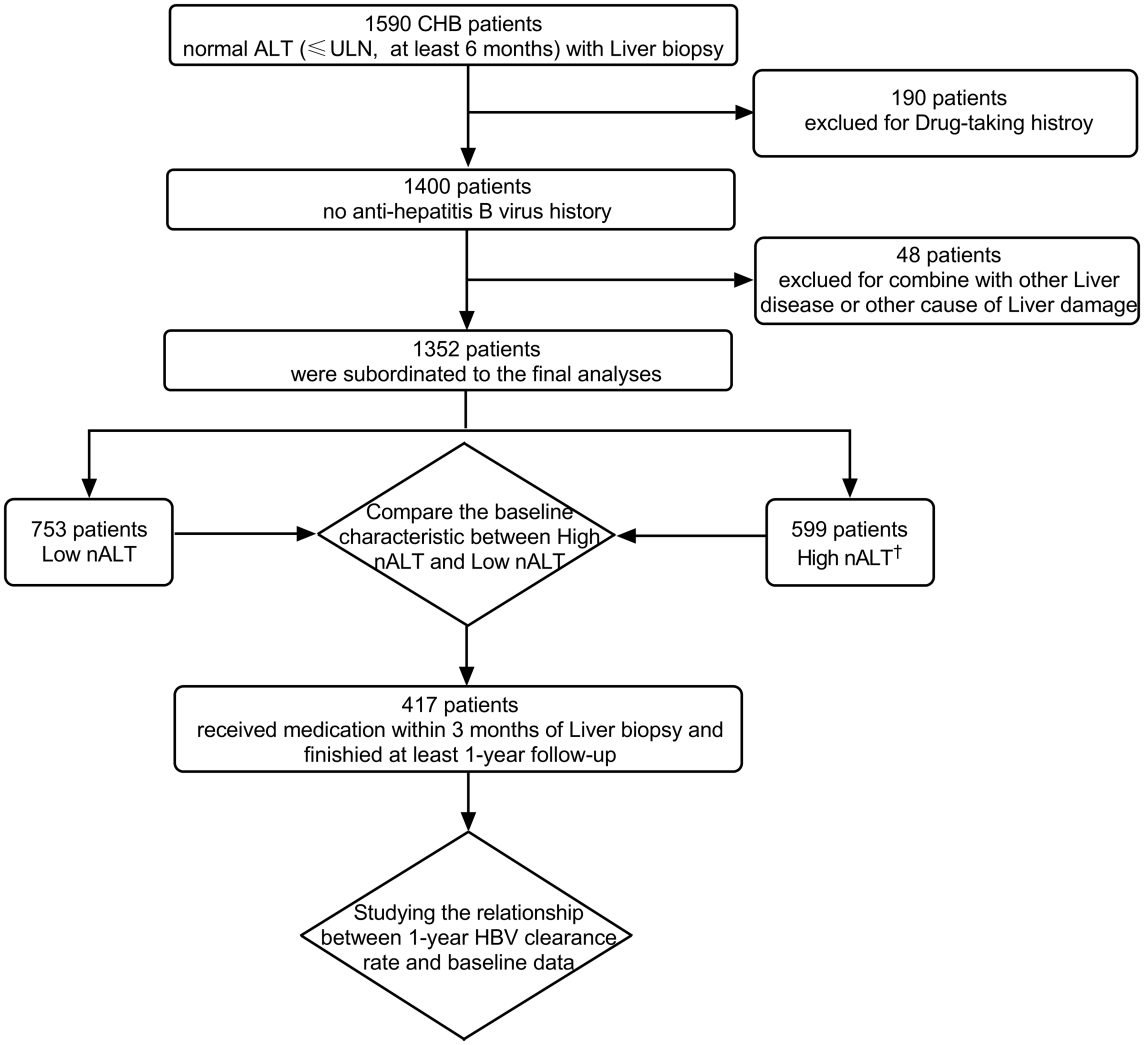
Flow diagram of study patients and reason of exclusion. †: High nALT of Male>30; High nALT of Female>19. HBV, hepatitis B virus; ULN, upper limit of normal; CHB, chronic hepatitis B; nALT, normal alanine aminotransferase.

A retrospective analysis was done of 1590 patients who underwent liver biopsy at the first affiliated hospital of Wenzhou medical university from January 2017 to December 2021. The study population consisted of HBsAg-positive who had normal ALT (≤ ULN, 40 U/L) for at least 6 months. All patients had been histologically diagnosed through liver biopsy. We excluded co-infection of HBV and HIV, HCV or HDV, or infection with EBV and CMV; patients with schistosomiasis liver disease or Wilson disease; patients who had received antiviral therapy; patients with alcoholic liver disease (alcohol consumption ≥ 40 g/day for male and ≥ 20g/day for female); and patients with other causes of liver damage (non-alcoholic fatty liver, drugs, autoimmune hepatitis, etc.). A total of 1352 patients were eligible for this study. We collected the follow up data of those opted for antiviral treatment based on their real-world preferences regardless of the type of antiviral regimen. Of these, 471 patients entered the next step of multivariate logistic regression analysis for HBV seroconversion (**Figure 1**).

### Ethics approval and consent statement

This study was conducted in accordance with the Declaration of Helsinki of 1975 and approved by the Research Ethics Committees of the first affiliated hospital of Wenzhou medical university(KY2022-158). Informed consent was obtained from all subjects included in the study. Written informed consent was obtained by each patient before liver biopsy.

### Laboratory tests

All the laboratory, histological, imaging and other clinical data were retrospectively collected using a structured data frame by review of medical records. A liver biopsy was recommended for CHB patients to determine the inflammation grades and fibrosis stages to assist in the decision for the initiation of antiviral treatment. The liver histological assessment of liver inflammation grade and fibrosis stage of each section was based on the Ishak or HAI score (which scoring system to choose depends on the scoring criteria set by the hospital at that time) by two experienced pathologists who were blinded to the clinical and biochemical data of patients. The laboratory tests before liver biopsy during hospitalization were defined as baseline.

### Statistical analysis

Continuous variables were expressed as the median and inter quartile range (IQR), whereas categorical variables were shown as counts and percentages. Continuous variables were compared using independent-group Student’s t tests or the Mann–Whitney U tests and categorical variables were compared using chi-squared tests. The optimal cut-off values were determined by Youden’s index. The factors predicting HBV DNA 1-year clearance were identified by multivariate logistic regression analysis. Variables with a p value of<0.1 in univariate analysis were selected into multivariate analysis by input step. Evaluating the predictive ability of different models by calculating the area under the ROC curve. All statistical analyses were carried out using the software program SPSSv26.0 (IBM Corporation). A two-tailed p value of<0.05 was considered statistically significant.

In this study, APRI is calculated by $\text{APRI}=\frac{\text{AST (IU/L)/ULN of AST}}{{\text{PLT (10}}^{9}\text{/L)}}\times 100$ , FIB-4 is calculated by $FIB-4=\frac{\text{Age (years)}\times \text{AST(IU/L)}}{{\text{PLT (10}}^{9}\text{/L)}\times \sqrt{\text{ALT (IU/L)}}}$ , GPR is calculated by $\text{GPR}=\frac{\text{GGT (IU/L)/ULN of GGT}}{{\text{PLT (10}}^{9}\text{/L)}}\times 100$ , AAR is calculated by $\text{AAR}=\frac{\text{AST}}{\text{ALT}}$ . In the formula, the ULN means upper limit of normal value.

## Results

### Significant variability in clinical features among patients grouped by baseline ALT according to AASLD 2016 ULN

**Table 1** shows the baseline characteristics of the study patients. According to ULN level of AASLD 2016, 753 patients (55.70%) had a low normal ALT (male ≤30U/L and female ALT ≤19U/L) and 599 patients (44.30%) had a high normal ALT (male >30U/L and female ALT > 19U/L). The two groups did not differ significantly in platelets (PLT) or total bilirubin (TBL). The high nALT group had a significantly higher proportion of female patients, HBsAg≥1000 and HBeAg positivity than the low nALT group (58.43% *vs* 39.71%, p< 0.001; 77.13% *vs* 66.27% p< 0.001; 44.07% *vs* 32.54% p< 0.001). The high nALT group also had a significantly higher Age, HBV DNA, γ-glutamyl transpeptidase (GGT) and aspartate transaminase (AST) level than the low nALT group (41.32 *vs* 39.69, p = 0.002; 5.20 *vs* 4.41 log10 IU/mL, p< 0.001; 25.79 *vs* 21.44 U/L, p< 0.001; 29.35 *vs* 22.80 U/L, p< 0.001). In the comparison of the current scoring systems, the high nALT group had a higher APRI, FIB-4 and GPR score (0.38 *vs* 0.29, p< 0.001; 1.17 *vs* 1.08, p = 0.009; 0.30 *vs* 0.24, p<0.001), while the AAR score of the high nALT group is lower (0.99 *vs* 1.28, p< 0.001). **Additional file 2** illustrates the violin plot of HBV DNA and GGT.

**Table 1:** Baseline clinical characteristics of all patients.

	Low nALT 753	High nALT^†^ 599	*P*-value
Age, years	39.69 ± 9.16	41.32 ± 9.89	0.002
Sex, n (%)			<0.001
Female	299 (39.71%)	350 (58.43%)	
Male	454 (60.29%)	249 (41.57%)	
HBV DNA (log IU/mL)	4.41 ± 2.65	5.20 ± 2.53	<0.001
HBsAg, n (%)			<0.001
≥ 1000	499 (66.27%)	462 (77.13%)	
<1000	254 (33.73%)	137 (22.87%)	
HBeAg, n (%)			<0.001
negative	508 (67.46%)	335 (55.93%)	
positive	245 (32.54%)	264 (44.07%)	
PLT	214.38 ± 54.05	213.33 ± 61.67	0.738
TBL	11.70 ± 5.85	11.21 ± 5.86	0.123
AST	22.80 ± 5.34	29.35 ± 9.10	<0.001
GGT	21.44 ± 13.90	25.79 ± 19.02	<0.001
APRI	0.29 ± 0.12	0.38 ± 0.20	<0.001
FIB-4	1.08 ± 0.56	1.17 ± 0.74	0.009
AAR	1.28 ± 0.40	0.99 ± 0.32	<0.001
GPR	0.24 ± 0.19	0.30 ± 0.26	<0.001
Liver damage^¶^			<0.001
non-significant	423 (56.18%)	261 (43.57%)	
significant	330 (43.82%)	338 (56.43%)	
Necroinfammation n (%)			<0.001
non-significant	472 (62.68%)	292 (48.75%)	
significant	281 (37.32%)	307 (51.25%)	
Fibrosis n (%)			0.001
non-significant	598 (79.42%)	430 (71.79%)	
significant	155 (20.58%)	169 (28.21%)	

Data are presented as a mean value with SD.

†: High nALT of Male>30; High nALT of Female>19; ¶: Liver damage (Necroinfammation or Fibrosis).

nALT, normal alanine aminotransferase; HBV DNA: Hepatitis B virus-deoxyribonucleic acid; HBsAg, hepatitis B surface antigen; HBeAg, hepatitis B e antigen; PLT, platelet; TBL, total bilirubin; AST, aspartate aminotransferase; GGT, gamma-glutamyl transferase; APRI, aspartate aminotransferase-to-platelet ratio index; FIB-4, fibrosis index based on four factors; AAR, AST/ALT ratio; GPR, gamma-glutamyl transpeptidase to platelet ratio.

### High normal ALT level strongly correlates with high liver histological disease rate

**Figure 2:**
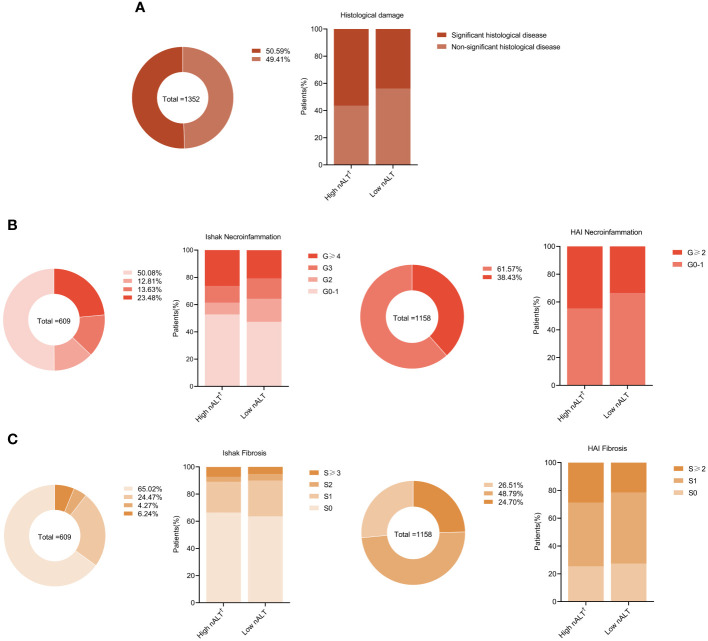
Distribution of liver histology of chronic hepatitis B patients in different baseline ALT. Proportions of liver significant histological disease **(A)**, inflammation grades **(B)**, and fibrosis stages **(C)** in study patients. †: High nALT of Male>30; High nALT of Female>19. nALT, normal alanine aminotransferase.

The proportion of patients with significant histological disease (significant liver necroinflammation or fibrosis) in the high nALT group was significantly higher than that in the low nALT group (56.43% *vs* 43.82%, p< 0.001, **Table 1**). Respectively, the proportion of significant liver necroinflammation and fibrosis also showed this trend (51.25% *vs* 37.32%, p< 0.001; 28.21% *vs* 20.58%, p = 0.001). As a retrospective study, the exact scoring system adopted for each patient relies on the scoring criteria picked by the hospital at that moment. We also presented the population distribution of different histological disease stages in different scoring systems, including necroinflammation and fibrosis (**Figure 2**).

### Clinical features and liver histological characteristics of patients with normal ALT followed after antiviral treatment

**Figure 3:**
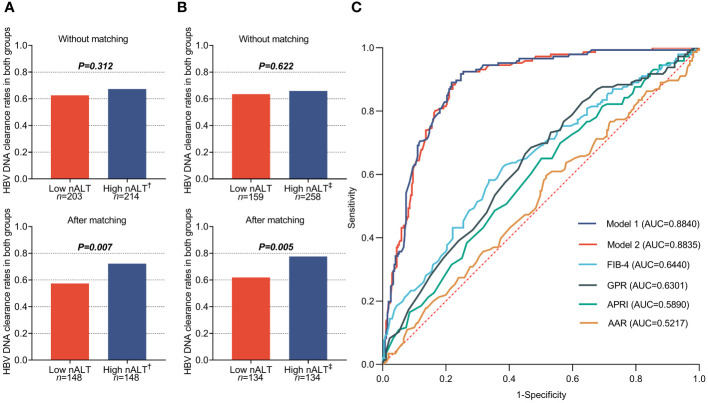
1-year HBVDNA clearance rate of chronic hepatitis B patients in different baseline ALT group **(A, B)**, and the receiver operating characteristic (ROC) curves **(C)** of different predict model based on the different High nALT cut-off level. †: High nALT of Male>30; High nALT of Female>19. ‡: High nALT of Male>29; High nALT of Female>15. Model 1: High nALT cut-off level defined as ALT of Male>29; ALT of Female>15; Model 2: High nALT cut-off level defined as ALT of Male>30; ALT of Female>19. nALT, normal alanine aminotransferase; AAR, AST/ALT ratio; APRI, aspartate aminotransferase-to-platelet ratio index; FIB-4, fibrosis index based on four factors; GPR, gamma-glutamyl transpeptidase to platelet ratio.

**Table 2** shows the baseline characteristics of the study patients with high normal ALT and low normal ALT who received antiviral treatment and followed up for at least one year. Out of 417 CHB patients, 203 patients (48.68%) had a low normal ALT (male ≤30U/L and female ALT ≤19U/L) and 214 patients (51.32%) had a high normal ALT (male >30U/L and female ALT > 19U/L). The two groups did not differ significantly in PLT, Ages, HBsAg, HBeAg, or HBV DNA 1-year clearance rate (**Figure 3A**). The high nALT group had a significantly higher proportion of female patients than the low nALT group (60.28% *vs* 47.29%, p = 0.008). The high nALT group also showed significantly higher HBV DNA and GGT level than the low nALT group (5.74 *vs* 5.21 log10 IU/mL, p = 0.017; 25.93 *vs* 22.16 U/L, p = 0.013). In the comparison of the current scoring systems, the high nALT group had a higher APRI and GPR score (0.41 *vs* 0.31, p< 0.001; 0.30 *vs* 0.25, p = 0.025), while the AAR score of the high nALT group is lower (1.01 *vs* 1.35, p< 0.001). The proportion of patients with significant histological disease in the high nALT group was significantly higher than that in the low nALT group (73.83% *vs* 55.17%, p< 0.001). Respectively, the proportion of significant liver necroinflammation and fibrosis showed the same trend (67.29% *vs* 46.80%, p< 0.001; 39.25% *vs* 25.62%, p = 0.003). **Table 3** also shows the baseline table re-grouped based on the cut-off values obtained from the Youden index.

**Table 2:** Baseline clinical characteristics of the study population according to AASLD 2016 criteria.

	Low nALT 203	High nALT^†^ 214	*P*-value
Age, years	39.63 ± 9.33	40.93 ± 9.70	0.166
Sex, n (%)			0.008
Female	96 (47.29%)	129 (60.28%)	
Male	107 (52.71%)	85 (39.72%)	
HBV DNA(Log IU/mL)	5.21 ± 2.35	5.74 ± 2.13	0.017
HBsAg, n (%)			0.179
≥ 1000	151 (74.38%)	171 (79.91%)	
<1000	52 (25.62%)	43 (20.09%)	
HBeAg, n (%)			0.083
negative	114 (56.16%)	102 (47.66%)	
positive	89 (43.84%)	112 (52.34%)	
PLT	210.34 ± 55.81	211.96 ± 63.99	0.784
TBL	11.98 ± 6.66	10.83 ± 4.91	0.045
AST	23.87 ± 6.73	31.22 ± 11.60	<0.001
GGT	22.16 ± 14.35	25.93 ± 16.41	0.013
APRI	0.31 ± 0.13	0.41 ± 0.23	<0.001
FIB-4	1.16 ± 0.62	1.24 ± 0.83	0.266
AAR	1.35 ± 0.44	1.01 ± 0.38	<0.001
GPR	0.25 ± 0.19	0.30 ± 0.22	0.025
Liver damage^¶^			<0.001
non-significant	91 (44.83%)	56 (26.17%)	
significant	112 (55.17%)	158 (73.83%)	
Necroinfammation n (%)			<0.001
non-significant	108 (53.20%)	70 (32.71%)	
significant	95 (46.80%)	144 (67.29%)	
Fibrosis n (%)			0.003
non-significant	151 (74.38%)	130 (60.75%)	
significant	52 (25.62%)	84 (39.25%)	

†: High nALT of Male>30; High nALT of Female>19.

¶: Liver damage (Necroinfammation or Fibrosis).

nALT, normal alanine aminotransferase; HBV DNA: Hepatitis B virus-deoxyribonucleic acid; HBsAg, hepatitis B surface antigen; HBeAg, hepatitis B e antigen; PLT, platelet; TBL, total bilirubin; AST, aspartate aminotransferase; GGT, gamma-glutamyl transferase; APRI, aspartate aminotransferase-to-platelet ratio index; FIB-4, fibrosis index based on four factors; AAR, AST/ALT ratio; GPR, gamma-glutamyl transpeptidase to platelet ratio.

**Table 3:** Study population characteristics based on youden’s index.

	Low nALT 159	High nALT^‡^ 258	*P*-value
Age, years	40.30 ± 9.56	40.30 ± 9.53	0.997
Sex, n (%)			<0.001
Female	59 (37.11%)	166 (64.34%)	
Male	100 (62.89%)	92 (35.66%)	
HBV DNA(Log IU/mL)	5.04 ± 2.31	5.75 ± 2.17	0.002
HBsAg, n (%)			0.062
≥ 1000	115 (72.33%)	207 (80.23%)	
<1000	44 (27.67%)	51 (19.77%)	
HBeAg, n (%)			0.032
negative	93 (58.49%)	123 (47.67%)	
positive	66 (41.51%)	135 (52.33%)	
PLT	206.46 ± 48.58	214.08 ± 66.11	0.209
TBL	12.30 ± 6.77	10.83 ± 5.13	0.012
AST	23.65 ± 6.41	30.11 ± 11.31	<0.001
GGT	22.89 ± 14.64	24.84 ± 16.04	0.213
APRI	0.31 ± 0.12	0.39 ± 0.22	<0.001
FIB-4	1.18 ± 0.62	1.20 ± 0.80	0.768
AAR	1.36 ± 0.44	1.06 ± 0.40	<0.001
GPR	0.27 ± 0.19	0.29 ± 0.22	0.318
Liver damage^¶^			0.036
non-significant	66 (41.51%)	81 (31.40%)	
significant	93 (58.49%)	177 (68.60%)	
Necroinfammation n (%)			0.007
non-significant	81 (50.94%)	97 (37.60%)	
significant	78 (49.06%)	161 (62.40%)	
Fibrosis n (%)			0.407
non-significant	111 (69.81%)	170 (65.89%)	
significant	48 (30.19%)	88 (34.11%)	

‡: High nALT of Male>29; High nALT of Female>15.

¶: Liver damage (Necroinfammation or Fibrosis).

Abbreviations as in **Table 2**.

### ALT ULN recommended by AASLD 2016 effectively differentiates HBV DNA 1-year clearance rate

Clinical features after PSM of patients with high normal ALT and low normal ALT who received antiviral treatment and followed up for at least one year is shown in **Table 4**. All the factors show no significant difference between two groups, except HBV DNA 1-year clearance rate (**Figure 3A**). **Table 5** also shows the re-grouped baseline table after PSM.

**Table 4:** Clinical characteristics of the study population according to AASLD 2016 criteria after matching all different baseline confounders between groups.

	Low nALT 148	High nALT^†^ 148	*P*-value
Age, years	39.09 ± 9.53	39.97 ± 9.43	0.422
PLT	210.82 ± 60.41	211.47 ± 63.13	0.927
TBL	11.84 ± 6.93	11.30 ± 5.21	0.455
Sex, n (%)			0.907
Female	76 (51.35%)	77 (52.03%)	
Male	72 (48.65%)	71 (47.97%)	
HBV DNA (Log IU/mL)	5.63 ± 2.31	5.35 ± 2.14	0.278
HBsAg, n (%)			0.782
≥ 1000	115 (77.70%)	113 (76.35%)	
<1000	33 (22.30%)	35 (23.65%)	
HBeAg, n (%)			0.561
negative	72 (48.65%)	77 (52.03%)	
positive	76 (51.35%)	71 (47.97%)	
GGT	23.62 ± 15.97	24.47 ± 11.45	0.599

†: High nALT of Male>30; High nALT of Female>19.

Abbreviations as in **Table 2**.

**Table 5:** Study population characteristics based on Youden’s Index after matching of all different baseline confounders between groups.

	Low nALT 134	High nALT^‡^ 134	*P*-value
Age, years	39.59 ± 9.79	40.61 ± 9.66	0.390
PLT	207.30 ± 49.84	212.60 ± 66.52	0.461
TBL	12.00 ± 6.56	11.41 ± 5.42	0.423
Sex, n (%)			0.711
Female	58 (43.28%)	55 (41.04%)	
Male	76 (56.72%)	79 (58.96%)	
HBV DNA (Log IU/mL)	5.18 ± 2.32	4.86 ± 1.98	0.226
HBsAg, n (%)			0.493
≥ 1000	100 (74.63%)	95 (70.90%)	
<1000	34 (25.37%)	39 (29.10%)	
HBeAg, n (%)			0.170
negative	75 (55.97%)	86 (64.18%)	
positive	59 (44.03%)	48 (35.82%)	
GGT	23.44 ± 15.55	25.15 ± 12.64	0.325

‡: High nALT of Male>29; High nALT of Female>15.

Abbreviations as in **Table 2**.

### High normal ALT is an independent predictor for 1-Year HBV DNA clearance

**Table 6** shows the factors associated with 1-year HBV DNA clearance. HBeAg positive, GGT and ALT value (OR 0.024, 95% CI 0.011–0.055, p< 0.001; OR 1.027, 95% CI 1.006–1.050, p = 0.013; OR 1.993, 95% CI 1.115–3.560, p = 0.020) were independent prediction factors. The factors associated with 1-year HBV DNA clearance after re-group based on the cut-off values obtained from the Youden index also shown in **Table 6**. Similarly, the HBeAg positive, GGT and ALT value (OR 0.025, 95% CI 0.011–0.056, p< 0.001; OR 1.028, 95% CI 1.006–1.050, p = 0.011; OR 2.000,95% CI 1.015–3.793, p = 0.034) were independent prediction factors. **Figure 3C** shows the AUC of these two models and the current scoring system. Model 1 and model 2 did not exhibit a large discrepancy in prediction ability (0.8840 VS 0.8835).

**Table 6:** Factors associated with the occurrence of HBV DNA clearance at 12-month follow-up.

	High nALT cut-off level defined as ALT of Male>30; ALT of Female>19			High nALT cut-off level defined as ALT of Male>29; ALT of Female>15		
	OR	95%CI	*P*-value	OR	95%CI	*P*-value
Sex						
Female	1.000			1.000		
Male	0.755	0.399-1.427	0.386	0.821	0.423-1.594	0.560
HBV DNA						
≥ 2000 IU/mL	1.000			1.000		
<2000 IU/mL	1.645	0.502-5.393	0.411	1.730	0.522-5.737	0.370
ALT^§^						
Low nALT	1.000			1.000		
High nALT	1.993	1.115-3.560	0.020	2.000	1.055-3.793	0.034
HBeAg						
negative	1.000			1.000		
positive	0.024	0.011-0.055	0.000	0.025	0.011-0.056	0.000
HBsAg						
≥ 1000	1.000			1.000		
<1000	0.615	0.282-1.340	0.221	0.626	0.287-1.367	0.240
GGT	1.027	1.006-1.050	0.013	1.028	1.006-1.050	0.011

§: Divided in two groups according to the different threshold of ALT.

nALT, normal alanine aminotransferase; HBV DNA: Hepatitis B virus-deoxyribonucleic acid; HBsAg, hepatitis B surface antigen; HBeAg, hepatitis B e antigen; GGT, gamma-glutamyl transferase.

## Discussion

The latest guidelines or recommendations for diagnosis and treatment of chronic hepatitis B infection issued by American Association for the Study of Liver Diseases (AASLD), European Association for the Study of the Liver (EASL) and Asian Pacific Association for the Study of the Liver (APASL) divide HBV infection into four phases (3–5). However, clinically, chronic hepatitis B patients have complex conditions, and a considerable proportion of chronic hepatitis B patients do not meet the diagnostic criteria of any particular phase and cannot be directly classified into them. Taking AASLD 2018 guidelines as an example, about 40% patients cannot be clearly assigned to one of the four phases and are in Grey Zone.

The Grey Zone is not an intermediate phase, patients can stay in Grey Zone for a long period and have a high risk of illness and death. A clinical study including 3366 patients found that after 10 years of follow-up, 52.7% of the patients who were originally in the Grey Zone were still in the Grey Zone (8). A 6-year follow-up study of 1465 patients with chronic hepatitis B found that patients with CHB in Grey Zone had a significantly higher risk of developing hepatocellular carcinoma than patients in immune tolerance and inactive stages (10). A subsequent longitudinal study spanning a decade, involving 1303 patients with Grey Zone CHB and 1370 with inactive CHB, revealed significant disparities. The cumulative hepatocellular carcinoma (HCC) incidence among those in the Grey Zone remained markedly elevated, at 4.5 times that of the inactive CHB cohort. Notably, after the 10-year follow-up, 857 patients persisted in the Grey Zone, with their cumulative HCC incidence surging to 9.6 times that of those transitioning into the inactive chronic stage (9). The above research challenges the current course of staging and treatment strategies of chronic hepatitis B.

Based on current research, 40U/L is not appropriate as the normal threshold of aminotransferase in CHB, because it does not accurately indicate having a normal liver. A study found that significant liver damage was detected in 40% of CHB patients with normal ALT and high HBV DNA upon biopsy (13). Another study has found that high-normal ALT is an indicator for liver histopathology in HBeAg-negative chronic hepatitis B (7). The study used 20U/L as the cut-off value to divide the CHB patient with normal ALT (<40U/L) into high normal ALT and low normal ALT groups. They found that a high value of normal ALT was a histopathological indicator, and the histopathological disease rate in the high normal ALT group was much higher than that in the low normal ALT group. However, studies have found that the normal cut-off value of transaminase in women patients with histopathological disease is lower than that in men (14–18). In this research, we retrospectively studied 1352 CHB patients with ALT ≤ 40U/L who underwent liver biopsy. According to AASLD 2016 recommendation male ALT>30U/L and female ALT>19U/L, they were divided into high normal ALT (nALT) and low normal ALT (nALT) groups. By comparing the histological disease status, HBV DNA, HBeAg and other clinical indicators of the two groups, we found patients in the high nALT group had higher HBV DNA, GGT and histological disease rates than those in the low nALT group (the difference was statistically significant), and 56.4% of them had significant histological disease (**Table 1**, **Additional file 2**). Moreover, the high nALT group had higher disease rates than the low nALT group, regardless of whether it was a single evaluation of histological inflammation or histological fibrosis (**Figure 2**). This suggests that these two groups are completely different CHB patient populations, and the high histological disease rate and high viral load in the high nALT group are problems that we cannot ignore. This also implies that the cut-off value of 40U/L is not suitable for CHB patients; using male>30U/L and female ALT>19U/L as standards can better reflect the liver inflammation situation of ALT “normal” patients under the current standard.

The virological response of patients in the high nALT group who had histological disease and higher viral load to antiviral treatment is an important issue that warrants our attention. Likewise, the treatment prognosis of the significant histological progressors in the low nALT group is also a matter of concern.

However, there is still a lack of research on the antiviral treatment effect of patients with ALT ≤ 40U/L, and the ALT cut-off values used in existing studies are not classified according to gender as recommended by AASLD. In this study, 417 patients opted for antiviral treatment based on their real-world preferences and completed at least one year of follow-up at our center. Like the baseline of the overall liver biopsy population, high nALT group had significantly higher HBV DNA, GGT and histological disease rates than low nALT group in this population (**Table 2**). In this study population, not all patients had histological disease; some chose antiviral treatment for other reasons such as family history of hepatocellular carcinoma or mother-to-child blockage. However, there was no significant difference in the one-year HBV DNA negative conversion rate among patients in our antiviral treatment population. Considering that ALT is related to other factors such as gender, we further used PSM to reduce bias. After PSM, high nALT group had a significantly higher one-year HBV DNA negative conversion rate than the low nALT group (**Figure 3A**).

Using the pre-liver biopsy baseline data in **Table 2**, we run multivariable logistic regression to predict the one-year HBV DNA clearance. The results showed that HBeAg (95% CI: 0.011-0.055), GGT (95% CI: 1.006-1.050), and ALT (95% CI: 1.115-3.560) were independent predictors of 1-year HBV DNA clearance in patients (**Table 6**). Patients in the high nALT group were more likely to achieve serological cure during antiviral therapy, which is an important finding that supports the current direction of expanding HBV antiviral therapy. A positive HBV DNA test result indicates the presence of infectivity, and viral replication can also lead to hepatocellular carcinoma. Additionally, a positive HBV DNA test result increases the likelihood of disease-related discrimination to varying degrees. Surprisingly, baseline HBV DNA levels were not independent predictors of 1-year HBV DNA clearance in patients. This supports The Chinese Prevention and Treatment of Chronic Hepatitis B Guideline (2019/2022) recommendation that HBV DNA positive is part of the indication for antiviral treatment regardless of HBV DNA value (11, 12). Not entirely consistent with what was suggested by the latest meeting of expanding CHB treatment is that in this study low nALT group patients did not benefit as much from antiviral treatment as high nALT group, suggesting that ALT is still one of the indicators that need to be referenced before antiviral treatment. But it seems 40U/L is not a suitable upper limit for normal values for HBV DNA patients. Not only this study, but a considerable number of studies have also suggested that patients still have a risk of histological disease and developing cirrhosis and HCC when the upper limit of normal values is set at 40 U/L (19, 20).

The cutoff values used in the above study were derived from the AASLD 2016 guidelines recommendation, which showed good discriminative ability. To determine whether 30 and 19 are appropriate ALT normal value limits, we calculated a new pair of cutoff values based on Youden’s index in this study population, male > 29, female > 15, to compare with the AASLD 2016 recommended cutoff values. Under the new cut off value, the two groups also had significant differences in baseline HBV DNA and histological liver disease (**Tables 2**, **3**). For histological fibrosis differentiation, this cutoff value was not as good as AASLD’s recommendation of 30 and 19. Similarly, we also performed PSM on the re-grouped data. After matching, there was a difference in 1-year HBV DNA clearance rate between the two groups (**Table 5**; **Figure 3B**). Similar to AASLD standards, further multivariate Logistic regression showed that HBeAg, GGT, and ALT were independent predictors of HBV DNA conversion at one year for patients (**Table 6**). We further compared our models with the current scoring system. By drawing ROC curves for comparison, both of our models had higher AUC level than the current scoring system. There was no obvious difference in area under curve between the two models (AUC:0.8840 *vs* 0.8835, **Figure 3C**). However, previous studies reported that the normal population ALT range was male 29-33U/L, female 19-25U/L (14–18). This study obtained female 16U/L which is not within this range. Its validity and accuracy still need more research to verify.

Moreover, we observed a difference in baseline GGT between two groups. Multivariate logistic regression analysis revealed that GGT was an independent predictor of HBV DNA clearance, even though most patients in our study had normal levels of GGT. This indicates that the hepatobiliary system status or unhealthy lifestyle habits may also influence HBV DNA clearance, albeit to a lesser extent but not insignificantly.

In conclusion, our study suggests that ALT 40U/L is not a suitable criterion for defining normal value for CHB patients. We recommend that male patients with ALT>30U/L and female patients with ALT>19U/L should undergo histological evaluation of liver disease as soon as possible. These patients are likely to have more favorable outcomes from antiviral therapy. Considering other factors such as preventing HBV DNA transmission in blood, suppressing HBV replication in liver to decrease HCC risk, and eliminating stigma associated with the disease, we propose that these patients should be offered antiviral therapy when feasible. Furthermore, we developed a predictive model to estimate the probability of HBV DNA clearance at one year, which can assist clinicians in making decisions about antiviral therapy for CHB patients.

## Data availability statement

The raw data supporting the conclusions of this article will be made available by the authors, without undue reservation.

## Ethics statement

The studies involving humans were approved by Research Ethics Committees of the first affiliated hospital of Wenzhou medical university. The studies were conducted in accordance with the local legislation and institutional requirements. The participants provided their written informed consent to participate in this study.

## Author contributions

CC: Conceptualization, Methodology, Writing – original draft. WS: Formal analysis, Methodology, Writing – original draft. EL: Methodology, Writing – original draft. YJ: Methodology, Writing – original draft. HC: Writing – original draft. KX: Methodology, Writing – original draft. LC: Writing – original draft, Methodology. RC: Writing – original draft, Conceptualization. YC: Writing – original draft, Formal analysis. JL: Writing – original draft, Funding acquisition. TC: Writing – original draft, Formal analysis. XL: Methodology, Writing – original draft. LZ: Writing – original draft, Methodology. NY: Writing – original draft. HZ: Writing – review & editing. ML: Conceptualization, Funding acquisition, Writing – review & editing.

## Supplementary material

Additional file 1 Phenotypes Defined by American Association for the Study of Liver Diseases 2018 Criteria by Practice Setting. £: ALT ULN levels were as follows: 35 U/L for males, 25 U/L for females. ALT, alanine aminotransferase; HBeAg, hepatitis B e antigen; HBV, hepatitis B virus; ULN, upper limit of normal.

Additional file 2 Violin plot of baseline GGT and Log10 HBVDNA levels between low nALT group and high nALT group. †: High nALT of Male>30; High nALT of Female>19. GGT, gamma-glutamyl transferase; nALT, normal alanine aminotransferase; HBVDNA: Hepatitis B virus-deoxyribonucleic acid.
