# Supplementary figures and images for: High normal alanine aminotransferase is an indicator for better response to antiviral therapy in chronic hepatitis B
Source: Front Immunol. 2024 Mar 14;15:1367265. doi: 10.3389/fimmu.2024.1367265 (PMC10973120; doi:10.3389/fimmu.2024.1367265)

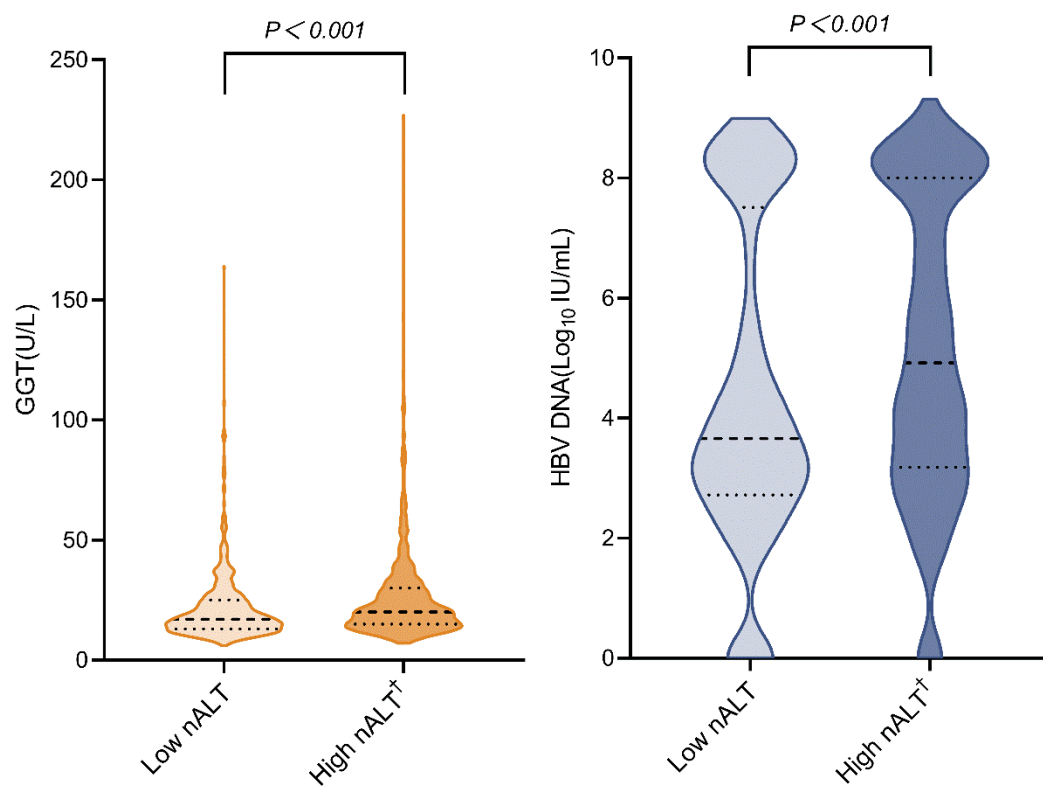

Supplement: Additional file 1 — Phenotypes Defined by American Association for the Study of Liver Diseases 2018 Criteria by Practice Setting. £: ALT ULN levels were as follows: 35 U/L for males, 25 U/L for females. ALT, alanine aminotransferase; HBeAg, hepatitis B e antigen; HBV, hepatitis B virus; ULN, upper limit of normal. [file Image_1.pdf]
