# Supplementary material for: High normal alanine aminotransferase is an indicator for better response to antiviral therapy in chronic hepatitis B
Source: Front Immunol. 2024 Mar 14;15:1367265. doi: 10.3389/fimmu.2024.1367265 (PMC10973120; doi:10.3389/fimmu.2024.1367265)
Supplement: Additional file 2 — Violin plot of baseline GGT and Log10 HBVDNA levels between low nALT group and high nALT group. †: High nALT of Male>30; High nALT of Female>19. GGT, gamma-glutamyl transferase; nALT, normal alanine aminotransferase; HBVDNA: Hepatitis B virus-deoxyribonucleic acid. [file Table_1.pdf]

	HBeAg	HBV DNA (IU/mL)	ALT[£] (U/L)	Liver biopsy
Immune tolerant Phase	+	>1×10^{6}	<1×ULN	Minimal inflammation, no fibrosis
Immune active Phase	+	≥2×10^{5}	≥2×ULN	Moderate or severe inflammation with or without fibrosis
Inactive Phase	-	<2000	<1×ULN	Absence of significant inflammation
Reactive Phase	-	≥2000	≥2×ULN	Moderate or severe inflammation with or without fibrosis
Indeterminate Phase	Any patient who does not fit the above criteria			
